# Ex-vivo investigation of probiotic bacterial adhesion to the intestinal mucus

**DOI:** 10.1016/j.heliyon.2024.e36339

**Published:** 2024-08-16

**Authors:** Thị-Thanh-Trúc Phùng, Sébastien Dupont, Laurent Beney, Sylvie Moundanga, Emmanuel Denimal, Phú-Hà Hồ, Thomas Karbowiak

**Affiliations:** a Université Bourgogne Franche-Comté, Institut Agro, Université de Bourgogne, INRAe, UMR PAM 1517, 1 Esplanade Erasme, 21000, Dijon, France; b Hanoi University of Science and Technology, School of Chemistry and Life Science, 1 Dai Co Viet Road, Hanoi, Viet Nam

**Keywords:** *Bacterial adhesion*, *Gastrointestinal tract*, *Ex-vivo approach*, *Probiotic efficiency*

## Abstract

Recent research has promoted considerable interest in the potential health benefits of the new generation of probiotics. Despite the abundance of probiotic supplements, their adhesion and thereby colonization in the intestinal tract of the host, a determining factor of probiotic efficacy, remains questionable. Indeed, the gastrointestinal tract, a multi-component and complex system, obscures the comprehensive understanding of the probiotic adhesion mechanism. This study aimed to investigate the adhesion capacity of probiotic bacteria using two ex-vivo approaches that were specifically developed to investigate the bacteria-mucus agglomeration and the viable adhesion to intestinal mucus. Five probiotic bacterial strains including *Escherichia coli*, *Lactiplantibacillus plantarum*, *Faecalibacterium duncaniae*, *Bifidobacterium longum*, and *Bifidobacterium longum* str. *infantis* were selected for the investigation. In that context, higher adhesion to mucus was demonstrated by *E. coli*, *L. plantarum*, and *B. infantis*, emphasizing strain-specific differences. While total agglomeration capacity ranged from 8 % to 82 %, actual viable adhesion to mucus remained rather low (0.6 %–2.9 %). SEM images revealed that morphological characteristics, chain and/or cluster forming ability, as well as the presence of surface exopolysaccharides, might have an impact on bacterial adhesion. This study contributes knowledge on probiotic adhesion as well as simple and effective ex-vivo approaches to investigate the bacterial adhesion to the intestinal mucus, which is prerequisite for further colonization in the gut of the host.

## Highlights

• Ex-vivo approaches to assess bacterial adhesion in the intestinal tract, considering the complexicity of the mucus.
• Viable bacteria–mucus adhesion index, a new concept of bacterial adhesion where the viability of bacteria is considered.
• Strong impact of the morphological attributes of bacteria on their adhesion potential to the intestinal surface.
• Positive effect of bacterial aggregation on their adhesion efficiency to extracellular matrices in the gut.

## Abbreviations

*E. coli*: *Escherichia coli* TG1K12
*L. plantarum*: *Lactiplantibacillus plantarum 103151T,* formerly known as *Lactobacillus plantarum 103151T*
*F. duncaniae*: *Faecalibacterium duncaniae A2-165,* formerly known as *Faecalibacterium prausnitzii A2-165*
*B. longum*: *Bifidobacterium longum* Reuter *BAA-*999TM
*B. infantis*: *Bifidobacterium longum* str.*infantis* Reuter 15697TM
DLVO theory: Derjaguin, Landau, Verwey and Overbeek theory
SI mucus: Small Intestinal mucus
LI mucus: Large Intestinal mucus
EPS: Exopolysaccharides
EMS: Extracellular Matrix Scaffold

## 1 Introduction

The phenomenon of bacterial adhesion can lead to advantageous or adverse effects depending on the health impacts of the bacterial strain. In the context of probiotics, defined as “live microorganisms that, when administered in adequate amounts, confer a health benefit to the host” [1], bacterial adhesion is desirable. Probiotics were discovered in the early 20th century and have been thought to have the ability to modify the microbiota system to improve health [2]. Probiotics are claimed to provide several health benefits for consumers, including boosting the immune system, preventing diarrhea, intestinal inflammation and colon cancer, and producing health beneficial compounds [[3], [4], [5], [6]]. Various probiotic supplements have been widely commercialized nowadays. However, the effectiveness of using probiotic supplements still remains questionable [7]. One of the key factors in the efficiency of such products is the adhesion of probiotics in the colon of the host [8]. This is crucial for colonization and development in the gut, and exerting beneficial effects on the host.

The intestinal mucus is a complex mixture of different components including glycoproteins, polysaccharides, lipids, salts and proteins [9]. Moreover, the composition of the intestinal fluid is very different among individuals [10], which might potentially affect the overall adhesion potential of bacteria to mucus. Besides, biological interactions are postulated to originate from receptors on the surface of bacterial cells interacting specifically with certain substrates on the intestinal mucus layer, causing specific adhesion in certain cases [11,12]. The interaction between bacteria and some specific components of mucus such as Caco-2, mucin type 2, bovine serum albumin, etc. has also been investigated and used as an indicator for bacterial adhesion in the intestinal tract [[13], [14], [15]]. However, it has been observed that bacterial strains isolated from mammalian intestines preferentially bind mucus over other specific components [16]. Mucus, being a complex system, has the potential to exhibit specific adhesion to certain substrates through its constituent molecules [17]. Conversely, these molecules may also induce repulsion with other substances. Accordingly, it is essential to evaluate the adhesion of bacteria to the whole mucus matrix, where all its components are taken into account. The bacterial adhesion to fresh porcine intestinal mucus has been investigated previously using a mucus immobilization assay coupled with a fluorescent labeling quantification method [18]. However, the labeling technique does not allow for the detection of cultivable cells, which is critical for the effectiveness of probiotics. Surface plasmon resonance (SPR) has also been used to investigate the adhesion of bacteria to mucin. This technique is a labeling-free detection and provides real-time data on the interaction process, allowing for the observation of dynamic changes [19]. The only drawback of this method is that it requires a SPR spectroscopy, which is costly and not always accessible. In another study, atomic force microscopy (AFM) has been used to assess the interaction of the mucus-binding protein from *Lactobacillus reuteri* to mucin [20]. This approach allows for the investigation of the specific interaction between the proteins present on the bacterial cell surface and mucus components such as mucin in a nanoscale. However, this method does not consider the complexity of the mucus composition.

In this context, this study aimed to elucidate the adhesion phenomenon of probiotic bacteria in the gastrointestinal tract using two ex-vivo approaches: bacteria-mucus agglomeration and viable adhesion to intestinal mucus. The agglomeration test in this research was originally introduced, while the adhesion of bacteria to the intestinal mucus layer was adapted from previous studies with major modifications. The global approach is based on the use of fresh intestinal mucus in order to take into account the complexity of this biological system. The viable adhesion assay applies the colony forming units (CFU) method to investigate the cultivability of bacteria in the intestinal mucus environment. These two methods were adopted as they are easily transposable to any laboratory and do not require any specific instruments. In addition, the observation of bacterial adhesion in the intestinal surface based on scanning electron microscopy also served as additional evidence on the interpretation of the adhesion indexes of the ex-vivo approaches. This approach aims to provide a better understanding as well as comprehensive and convenient methods for the evaluation of bacterial adhesion to intestinal mucus.

## 2 Materials and methods

### 2.1 Bacterial cultures

*Escherichia coli* strain TG1K12 was obtained from the Microbiology Laboratory Culture Collection, L'institut Agro Dijon, France. *E. coli* was grown at 37 °C, under continuous shaking at 150 rpm in Tryptic Soy Broth (22092 – Sigma Aldrich) medium under aerobic conditions.

*Lactiplantibacillus plantarum* strain 103151T previously named as *Lactobacillus plantarum* 103151T was obtained from the Collection of Pasteur Institute, Paris, France. *L. plantarum* was grown at 37 °C, under continuous shaking at 150 rpm in MRS medium (84613.0500 - VWR International S.A.S. - Fontenay-sous-Bois, France) under aerobic conditions.

*Bifidobacterium longum* Reuter BAA-999TM – Strain designation BB536 and *Bifidobacterium longum* subsp. *infantis* Reuter 15697TM – strain designation S12 were grown in modified reinforced clostridium broth medium (BK094HA – Biokar Diagnostics – Allonne, France) at 37 °C, inside an anaerobic chamber (Bactron I-2, Sheldon Manufacturing, USA) filled with 85 % N_2_, 5 % H_2_ and 10 % CO_2_ (v/v/v). All media and buffers were previously degassed.

*Faecalibacterium duncaniae* A2-165 previously named as *Faecalibacterium prausnitzii* A2-165 (DSM17677 – DSMZ- German Collection of Microorganisms and Cell Cultures GmbH - 38124 Braunschweig - Germany) were grown in LYHBHI (Brain-Heart-Infusion medium supplemented with 0.5 % yeast extract, 1 g/L cellobiose, 1 g/L maltose, 0.5 g/L L-cysteine, and 5 mg/L hemin) under anaerobic condition following the protocol of Raise 2020 [21].

The fresh cells were collected at the end of the exponential phase of the growth. Then they were washed 3 times with PBS (P4417 - Sigma Aldrich – Saint-Quentin - Fallavier) by centrifugation at 3000 G for 6 min.

### 2.2 Preparation of mucus and intestinal samples from pig intestine

In this study, pig intestines and mucus were used as an alternative to human ones due to their similarity in anatomic features [22]. Pig digestive systems were collected at the local abattoir (La Viande Naturellement – Lons-le-Saunier, 39570 Perrigny, France) on the day of slaughter. Since the animals were killed for commercial meat production, no ethical permit was required for the current study. The animals were fasted for around 12 h before slaughtering, with water available as needed, in accordance with the abattoir's regular operating procedures.

The mucus from the various parts of the pig intestine (small and large intestines) was collected in different containers, and then sieved through a mesh with a pore size of 0.4 mm to first remove large particles. The collected viscous fluid was diluted 2 times in 10 mM HEPES (4-(2-hydroxyethyl)-1-piperazineethanesulfonic acid) (15630080 – Thermo Scientific - Waltham, MA 02451, USA), centrifugated to remove water from the supernatant and separate from other impurities. The mucus was collected in a sterile jar and stored at −80 °C for further use.

### 2.3 Agglomeration of bacteria with intestinal mucus: ex-vivo assay

**Fig. 1:**
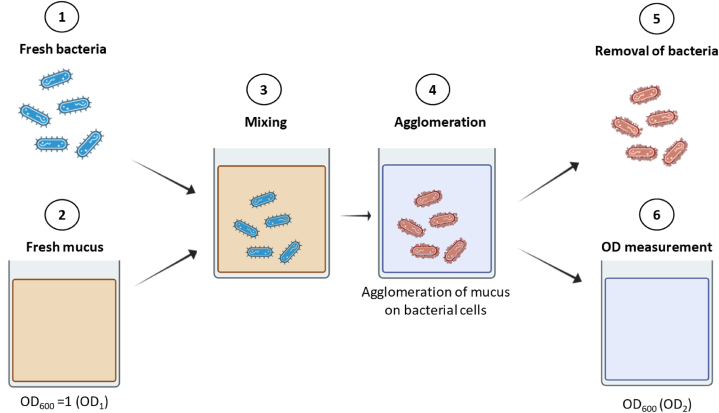
Schematic illustration of the agglomeration test of bacterial cells with intestinal mucus. **(1)** Fresh bacteria collected at the end of the exponential phase. **(2)** The fresh mucus was collected from the porcine intestinal tract, centrifugated to remove big particles, and diluted to OD_600_ = 1.0 (OD_1_). **(3)** Bacteria and mucus were vortexed for 1 min and **(4)** left for 15 min to allow agglomeration to occur. **(5)** The agglomerated bacteria were then separated from the supernatant by centrifugation. **(6)** The optical densities of mucus before (OD_1_) and after (OD_2_) agglomeration were used to calculate the bacteria – mucus agglomeration index according to Eq. (1) in order to normalize to 10^8^ CFU.

The agglomeration test of bacterial cells with intestinal mucus, developed in the present work, is illustrated in Fig. 1. Depending on the oxygen sensitivity of each strain, the experiment was performed under anaerobic or aerobic conditions, at room temperature (20 ± 1 °C). On the one side, the fresh bacterial culture was diluted with PBS to OD_600_ = 1.0 using a UV-VIS GeneQuant 1300 spectrophotometer (GE Healthcare Europe GmbH - Vélizy-Villacoublay, France), where the cells were initially counted and referred to as *n* (CFU/mL). On the other side, mucus was diluted 5 times with PBS in Eppendorf tubes, then centrifugated using the Eppendorf minispin (Eppendorf AG, Barkhausenweg 1, 22339 Hamburg, Germany) for 1 min at 3000 rpm to remove sediments. The mucus supernatant obtained was diluted with PBS to OD_600_ = 1.0 (so-called OD_1_). 1 mL of bacterial suspension was centrifugated in an Eppendorf tube for 1 min at 3000 rpm. The supernatant was removed, and the pellet containing the bacterial cells was resuspended with 1 mL of mucus (OD_1_) prepared previously, shaking for 1 min using a vortex mixer (Fisher Scientific - Waltham, MA 02451, USA). The mixture was let to agglomerate for 15 min before being centrifugated again for 1 min. The optical density of the supernatant was measured at the wavelength of 600 nm (so-called OD_2_). The agglomeration capacity of bacterial cells with mucus, referred to as the bacteria–mucus agglomeration index, was calculated based on the following equation, in order to normalize to 10^8^ CFU:

(1) $$Bacteria\;–\;mucus\;agglomeration\;index\;\left(\%\right)=\left(1\;‐\;\frac{{OD}_{2}}{\;{OD}_{1}}\right)\;x\;\frac{10^{8}}{n}\;x100$$

### 2.4 Bacterial adhesion on a mucus layer: ex vivo assay

**Fig. 2:**
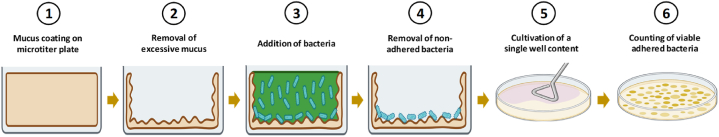
Schematic illustration of the ex-vivo assay developed to evaluate bacterial adhesion on a mucus layer. (1) Mucus was coated on a 96-well microtiter plate overnight. (2) The excessive mucus was removed, and the adhered mucus was fixed to the plate. The fresh bacterial culture was prepared, where the corresponding number of cells was determined. (3) Bacteria were then added to the mucus-coated wells, then letting adhesion to occur. (4) The non-adhered bacterial cells were then removed. (5) The whole content of each well was grown on MRS agar media. (6) The number of viable bacteria adhered to the mucus layer was count and normalized by subtracting the number of bacteria initially present in mucus, according to Eq. (2).

This bacterial adhesion test performed on a mucus-coated microplate was adapted from the study of [23] with major modifications [23]. Depending on the oxygen sensitivity of each strain, the experiment was carried out under anaerobic or aerobic conditions at 37 °C. The successive steps of the protocol are illustrated in Fig. 2. Porcine intestinal mucus was used instead of human one. The process of mucus preparation was described in section 2.2. First, 200 μL of small or large intestinal mucus were deposited in each well of a 96-well microplate (MAXISORP 439454 - Thermo Scientific – Roskilde, Denmark) and incubated for 12 h at 4 °C. Then, the excessive mucus was poured off from the plate. The adhered mucus was fixed to the plate by incubating at 65 °C, for 20 min. The wells were washed 3 times with 200 μL PBS/well. 100 μL of fresh bacterial culture (OD_600_ = 1.0, with the number of bacteria identified as *n*_*o*_) was added to each mucus-coated well. The plate was shaken using a microtiter plate orbital shaker Grant Bio PHMP (Keison Products – Essex, England) for 10 min and incubated at 37 °C for 1 h. The non-adhered bacteria were finally removed by washing with 100 μL PBS/well.

In order to assess the viability of the adhered bacteria fraction, the whole content of each well was taken by adding 100 μL PBS/well, mixing, picking and spreading on the specific agar media of the bacterial strain tested. The viability assessment was reported based on the number of colonies found on the agar plates (*n*_*1*_). A control sample was tested by replacing the bacterial culture with PBS solution. This allows the number of natural bacteria presenting in mucus to be determined (*n*_*blank*_). The adhesion of viable bacteria to the deposited mucus layer referred to as the viable bacteria–mucus index was calculated according to the following equation:

(2) $$\text{Viable}\;\text{bacteria}\;-\;\text{mucus}\;\text{adhesion}\;\text{index}\;\left(\%\right)=\frac{n_{1}-n_{blank}}{n_{o}}\;x\;100$$

### 2.5 Morphological study of selected bacterial strains based on SEM

The fresh bacterial culture, prepared following the protocol detailed in section 2.1 (and standardized at OD_600_ = 1.0), was deposited on a silicium wafer plate and was let to adhere for 5 min. The sample was washed 2 times with PBS, and was fixed with a solution containing paraformaldehyde 4 % (v/v) and glutaraldehyde 2.5 % (v/v) for 24 h. Then, the samples were subjected to several washing steps with increasing ethanol percentages of 30, 50, 70 and 90 % (v/v water) in order to progressively remove water (washing was performed 2 times for each concentration with 20 min for each washing step). The remaining water content was eliminated by critical point drying in liquid CO_2_ (EM CPD030, Leica microsystems, Austria). The dry samples were examined using a FEG-SEM Hitachi (SU8230, Hitachi, Japan), at an accelerating voltage of 3 kV (equipment available at DImaCell Platform, INRAE Dijon), after being sputter coated with a thin gold layer (5 nm) under vacuum, using an ion sputter coater (Q150T ES plus, Quorum, UK).

### 2.6 Ex-vivo investigation of bacterial adhesion to the intestinal epithelium based on SEM

The porcine intestine was collected as previously described in section 2.2. The intestine was cut into small pieces of 1 × 1 cm^2^. The large particles and the impurities were removed by washing with HEPES 10 mM. The bacterial culture, prepared following the protocol detailed in section 2.1 (and standardized at OD_600_ = 1.0), was deposited on the intestinal surface and was let to adhere for 5 min. The samples were washed 2 times with HEPES 10 mM, then fixed, washed with ethanol, dried using a critical point drying system, gold coated and observed with SEM, as previously described in section 2.5.

### 2.7 Statistical analysis

The data were presented as the mean ± standard deviation, based on a minimum of three independent replicates. Statistical comparisons of the means were conducted utilizing one-way analysis of variance (ANOVA), followed by Tukey's post hoc test. The Student's t-test was employed to compare the means between the two data sets of the same strains. The significance levels for all the tests were set at 95 % confidence (p < 0.05). Data processing was performed using MATLAB.

### 2.8 SEM image treatment

The raw images, obtained by scanning electron microscopy, underwent digital post-processing using Adobe Photoshop software (Adobe Inc. 2023) retrieved from https://www.adobe.com/products/photoshop.html. The 8-bit grayscale images were converted to 8-bit RGB color images. A clipping mask was defined semi-automatically for each bacterium, and a simple color filter was applied to each image using this mask. This processing aimed to highlight the bacteria in context by colorizing them. During this process, no pixels were added or removed, only the color hue was affected.

## 3 Results and discussion

### 3.1 Agglomeration of bacteria to the intestinal mucus

The interaction with intestinal mucus can be seen as the first step in the colonization process of bacteria in the gut. In this regard, the method proposed to study the agglomeration of bacteria to intestinal mucus was developed for the first time in the present work. The use of fresh intestinal mucus allows the complex composition of intestinal fluid to be integrated into the investigation of the adhesion behavior of bacteria.

**Fig. 3:**
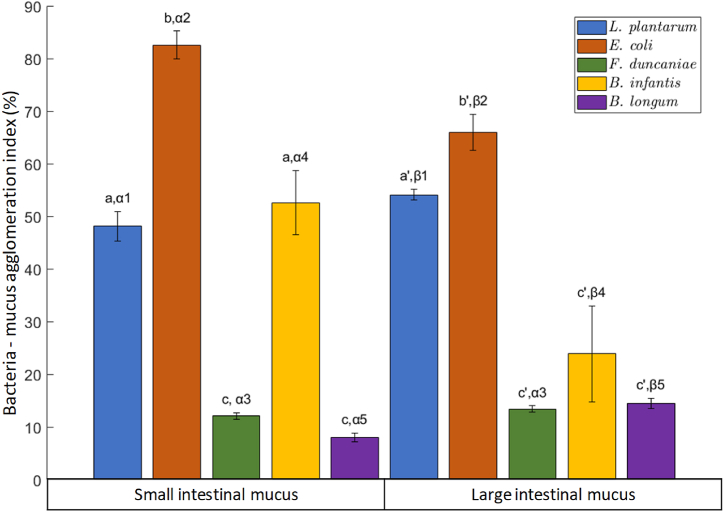
Agglomeration of bacteria with intestinal mucus referred to as bacteria – mucus agglomeration index. For each bacterial strain placed in contact with fresh mucus, the experiments were performed at a normalized concentration. The Latin letters a, b and c distinguish the difference between the 5 strains for their agglomeration ability with small intestinal mucus using one-way ANOVA with Tukey's test (p < 0.05). Similarly, the Latin letters a', b' and c' refer to their agglomeration ability with the large intestinal mucus. The difference between the agglomeration of each strain with SI and LI mucus was further analyzed by the student's t-test (p < 0.05). Corresponding results are indicated by the Greek letters α and β, followed by a number distinguishing each of the 5 strains.

Fig. 3 displays the agglomerating capacity of bacteria with mucus, previously collected from the small or large intestine of pig. The corresponding values ranged from 8 % to 82 %. *E. coli* exhibited a significantly higher agglomeration capacity with intestinal mucus compared to other strains, followed by *L. plantarum*. The same superior agglomeration of *E. coli* and *L. plantarum* was observed with both small and large intestinal mucus. In contrast, *B. longum* and *F. duncaniae* showed similarly low agglomeration capacity with mucus from both small and large intestines. Their corresponding agglomeration index falls below 15 %, which is 3–4 times lower than that of *E. coli* or *L. plantarum*. Comparing the agglomeration capacities of the same strain to 2 different types of mucus, *L. plantarum*, *E. coli* and *B. infantis* displayed different agglomeration capacities between SI and LI mucus, while it remained similar for *F. duncaniae* and *B.longum*.

*Bifidobacterium*, *Lactobacillus*, and *Faecalibacterium* are typical bacterial genera in the mucus-associated community [11,[24], [25], [26], [27]]. Thus, the agglomeration capacity of these strains with intestinal mucus was expected to be all high. However, according to this ex-vivo assay, each strain exhibited a very different behavior when placed in direct interaction with intestinal mucus. There can be various factors affecting the agglomeration index of bacteria to intestinal mucus. One of them might be the viability of bacterial cells. To investigate this hypothesis, the viable adhesion test of probiotics on the mucus layer was performed and the results are reported in section 3.2. Furthermore, the SEM observation was performed to further investigate the impact of bacterial morphological properties on their adhesion behavior in the intestinal tract (the corresponding results are reported in section 3.3).

### 3.2 Bacterial adhesion on intestinal mucus layer

The mucus blanket is nearly impermeable to microorganisms under physiological conditions. This means that bacteria can only adhere to the outer layer of the mucus barrier but not directly to the epithelial surface [28]. For this reason, in this study, the ability of bacteria to adhere to a mucus layer was performed. Previously, the bacterial adhesion potential to the gut has been widely investigated through the adhesion to some certain intestinal epithelial cell lines or proteins, for example Caco-2, BSA, MUC2, etc. [15,29,30]. In the present work, in order to take into account the impact of all the components existing in mucus to the bacterial adhesion, mucus was directly used to consider the full complexity of this biological environment. Regarding the counting method, previous studies have been using labeling techniques for the enumeration of bacterial cells, which could thus potentially include both dead and alive cells. However, in this study, the CFU method was applied to quantify the healthy and reproducible probiotic cells.

**Fig. 4:**
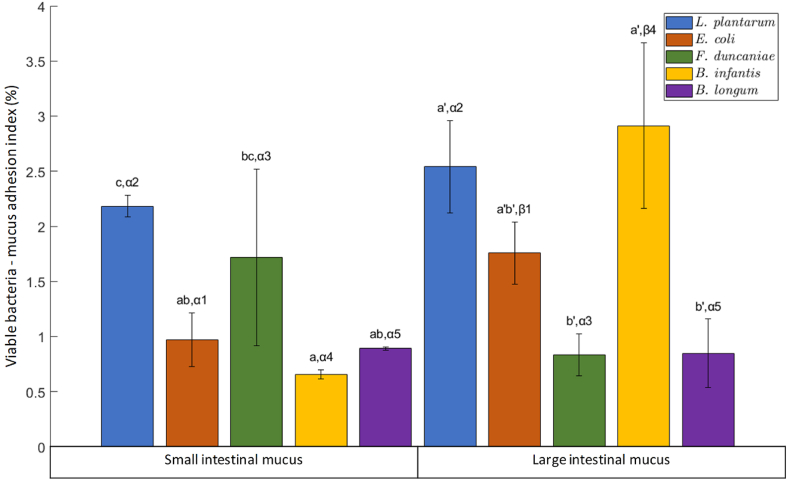
Adhesion of viable bacteria to intestinal mucus layer coated on microtiter plate referred to as viable bacteria – mucus adhesion index. The Latin letters a, b and c distinguish the difference between the 5 strains for their adhesion to small intestinal mucus using one-way ANOVA with Tukey's test (p < 0.05). Similarly, the Latin letters a', b' and c' refer to their adhesion to the large intestinal mucus. The difference between the adhesion of each strain to SI and LI mucus was further analyzed by the student's t-test (p < 0.05). Corresponding results are indicated by the Greek letters α and β, followed by a number distinguishing each of the 5 strains.

Fig. 4 displays the adhesion of viable bacteria to an intestinal mucus layer coated on a microtiter plate. The adhesion of *L. plantarum* and *F. duncaniae* to SI mucus was better than that of the other 3 strains. In contrast, regarding the adhesion to LI mucus, *L. plantarum*, *E. coli* and *B. infantis* displayed a higher ability to adhere compared to the other two strains. Moreover, considering the adhesion index of each strain to SI and LI mucus, there was no difference in the adhesion index of *L. plantarum*, *F. duncaniae* and *B. longum* to these two different types of mucus. As a general consideration, it is worthy to note that the number of alive bacteria adhering to the mucus was surprisingly low for all strains, less than 3 % of the total quantity of initial cells. This is in agreement with the result obtained from a previous study by Kainulainen et al., where the adhesion of *Lactobacillus* strains to intestinal mucus was also reported to be very low, less than 3 % [31]. However, the counting technique used in their study was not the same. In their case, bacterial cells were radioactively labeled in order to quantify them based on radioactive signals [[31], [32], [33]]. In such case, the labeling step usually causes lethal effects to bacterial cells and potentially modifies the cell surface properties, also leading to changes in the adhesion behavior of bacteria. Therefore, the actual adhesion efficiency might differ from the results obtained in those studies. In the present study, bacteria were cultured and quantified directly instead of labeling. This avoids aggressive impacts on bacterial cells and preserves the cell surface properties. Furthermore, this approach demonstrates the ability of bacteria to adhere in a strong viable state, which is the expectation of probiotic supplementation. In comparison with the agglomeration capacity of these strains with mucus, which ranged in 8–82 %, as previously reported in section 3.1, the adhesion of viable cells was much lower. The difference between the results of the two experiments was likely due to the viable status of the bacteria since the present adhesion test considered only viable cells that can further grow on agar media, while the previous agglomeration test did not distinguish between agglomeration phenomenon involving both viable and non-viable bacteria. However, some correlation exists between the results from the agglomeration test and the adhesion test. *E. coli* and *L. plantarum* exhibited in both cases better interactions with mucus compared to *F. duncaniae* and *B. longum*. To clarify the relevance and discrepancy between the results of these two tests, the effects of the viable state on bacterial cell surface properties and thereby on their adhesion to mucus should further be studied.

### 3.3 Bacterial adhesion on the intestinal surface

In order to further investigate the effect of the bacterial morphology on their adhesion efficiency in the gastrointestinal tract, scanning electron microscopic observation was used as additional evidence, complementary to the two previous *ex-vivo* approaches discussed in this work.

Table 1 displays the morphological characteristics of the 5 selected bacterial strains. According to the SEM micrographs, all 5 strains appeared in rod shapes, belonging to the bacillus family. However, each strain possessed unique morphological features. *L. plantarum*, *E. coli* and *B. longum* cells tend to separate from other single cells while *F. duncaniae* and *B. infantis* cells aggregate with each other forming chains. The 5 strains exhibited different cell dimensions. The cell diameters ranged from 0.3 to 0.9 μm, while their lengths ranged from 1.1 to 1.9 μm, except for *F. duncaniae* which was found in long chains with variable lengths. *L. plantarum* was observed with smooth cell surfaces and slightly perpendicular ends. It displays cells having a length in the range of 1.2–1.9 μm and a diameter of about 0.5 μm. *E. coli* cells appeared with a rough surface. This could be due to the presence of pili or exopolysaccharides (EPS) on the outer layer of the cells. *E. coli* was about the same length as *L. plantarum* with a length of 1.3–1.9 μm and a thick diameter of 0.7–0.9 μm. *F. duncaniae* cells presented with smooth cell surfaces and round ends. It displays a unique morphological feature compared to other strains with long chain formations of varied lengths of 1.1–17.3 μm, with a diameter of around 0.5 μm. Lastly, although *B. longum* and *B. infantis* are classified as the same species, their morphological expressions are different from each other. *B. longum* cells possess an elongated shape, with perpendicular ends, while *B. infantis* cells are gourd-shaped, with rounded ends. *B. longum* cells are in general the same length as single cells of *B. infantis* (1.3–1.4 μm), but with a smaller diameter (0.3–0.4 vs. 0.5–0.7 μm, respectively). *B. longum* cells were found to aggregate in pairs of 2 cells. Both *B. infantis* and *B. longum* strains were observed to be covered by a layer of polysaccharides outside the cell.

**Table 1:** Morphological characteristics of the 5 bacterial strains: *Lactiplantibacillus plantarum*, *Escherichia coli*, *Faecalibacterium duncaniae*, *Bifidobacterium infantis* and *Bifidobacterium longum*. For SEM observation, fresh bacterial cultures collected at the end of the exponential phase of their growth were deposited and fixed on silicium wafer plates. The cell dimensions of bacteria were measured from SEM images. Each strain was measured randomly from 10 cells for their length and diameter. The min-max values were reported.

Strai	SEM image	Characteristic size of single-cell	
		Length (μm)	Width (μm)
*Lactiplantibacillus plantarum*		1.2–1.9	0.5–0.6
*Escherichia coli*		1.3–1.9	0.7–0.9
*Faecalibacterium duncaniae*		1.1–17.3	0.5–0.6
*Bifidobacterium longum*		1.3–1.4	0.3–0.4
*Bifidobacterium infantis*		1.3–1.4	0.5–0.7

Blank pig intestine samples were prepared for SEM investigation without depositing any bacteria. The observation confirmed that there was no residential gut microbiota on the surface of intestinal samples after the initial treatment with HEPES solution. Accordingly, it can be stated that the bacterial cells further observed with SEM belong to the strain of interest. In addition, it is noteworthy that after the initial treatment, the intestinal samples did not show any damage and retained their initial morphological state. Moreover, a layer of mucus was still present on the intestinal surface.

**Fig. 5:**
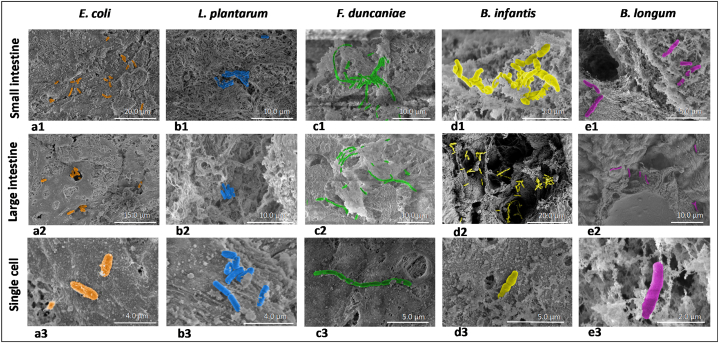
Scanning electron microscopic images of the 5 bacterial strains **a.***Escherichia. coli*, **b.***Lactiplantibacillus plantarum*, **c.***Faecalibacterium duncaniae*, **d.***Bifidobacterium longum infantis*, **e.***Bifidobacterium longum* adhered to the pig intestinal surface. Pig intestine samples were previously treated to remove the gut microbiota and impurities in order to keep a layer of mucus on the intestinal surface before the deposition of the bacteria of interest. The bacteria were harvested at the end of the exponential phase of growth. 1 mL of bacterial culture (OD_600_ = 1.0) was deposited on each piece of the intestine (1 × 1 cm^2^) and let to adhere for 5 min. The samples were then washed twice with 1 mL of PBS for each washing step before fixation and critical point drying steps. The number 1, 2 and 3 refer to the images of bacteria on small intestinal surface, large intestinal surfaces, and their single cells, respectively.

Fig. 5a1, a2 and a3 display the SEM images of *E. coli* adhered to the porcine intestinal surface. *E. coli* was the only of the 5 tested strains whose pili were clearly observed (Fig. 5a3). The presence of pili in *E. coli* has been reported in previous studies, typically with many different types [34,35]. These appendages play an important role as mechanosensors to initiate the formation of specific adhesion of the bacterial cells with the receptors on the intestinal surface [[35], [36], [37]]. The cells of *E. coli* were found separately distributed on the surface of the small and large intestinal surfaces (Fig. 5a1, 5a2). Therefore, specific adhesion caused by pili could be the main factor that helped *E. coli* cells to adhere more firmly to the intestinal epithelium. This assertion was correlated with the previous study by Pizarro-Cerdá and Cossart [38]. Furthermore, the presence of pili could be the reason for the higher agglomeration and adhesion efficiency of *E. coli* cells to intestinal mucus compared to other strains in the two previous *ex-vivo* experiments discussed in sections 3.1, 3.2. This might be caused by the existence of functional groups of the molecular structure of pili that help interact with specific substrates in the mucus, leading to the formation of multiple adhesion sites.

Fig. 5b1, b2 and b3 demonstrate SEM images of *L. plantarum* cells on the surface of the small and large intestines of pigs. *L. plantarum* cells were found concentrated on a network of biological macromolecules in the small intestinal surface (Fig. 5b1). This structure appeared similar to glycocalyx, composed of glycosaminoglycans, glycoproteins and glycolipids, which cover and modulate access to receptors on the surface of the small intestine [39,40]. This raises the hypothesis that specific adhesion occurred between the *L. plantarum* cells and the specific receptors on the network of glycocalyx, holding the cells against washing steps, which could represent the effect of bulk liquid flow in the intestinal cavity. On the large intestinal surface, *L. plantarum* tended to hide inside the intestinal crypts, which made it difficult to observe and quantify the total number of actually adhered cells (Fig. 5b2).

Fig. 5c1, c2 and c3 exhibit the SEM images of *F. duncaniae* cells adhered on the porcine intestinal surface. *F. duncaniae* was easily found in large quantities on both small and large intestinal surfaces. The bacterial cells clustered and formed chains with varied lengths. This morphological feature of *F. duncaniae* was also reported in previous studies [41,42]. In fact, the intestinal environment is a highly complex system. The presence of various macromolecules established a complex network as observed in Fig. 5c1 and 5c2. This might be a network of extracellular matrix scaffold (EMS) presenting on the intestinal surface, which consists of various components including structural proteins, glycosaminoglycans, matricryptic peptides, matrix-bound nanovesicles and growth factors [43]. Thus, despite the low rate of agglomeration and adhesion to the mucus layer compared to the other strains, as reported in the previous experiments, the formation of long chains is a significant factor that helps *F. duncaniae* cells to be trapped in the EMS network on the surface of the intestinal epithelium, and thus to finally adhere better. Also, this phenomenon could partly explain the low adhesion efficiency of *F. duncaniae* on a thin mucus layer without these specific structural features of the intestinal surface, as noticed in section 3.2. Since the quantification of single cells was based on culturing and counting colonies, the number of colonies detected was expected to accurately reflect the number of single cells. However, from a chain or a cluster, which consists of multiple cells, only a single colony is formed. Therefore, the actual number of single cells can be much higher than this underestimated number of colonies.

Fig. 5d1, d2 and d3 display the SEM images of *B. infantis* adhered on the porcine intestinal surface. *B. infantis* displayed T or Y shapes and blunted ends. This specific morphology of *B. infantis* has also been reported in a previous study [44]. Unlike *F. duncaniae*, *B. infantis* appeared on SEM images and did not form long chains. Instead, their short chains attached together in clusters. The cell surface of *B. infantis* was covered by a layer of film, which could represent EPS, helping them to strengthen the biofilm formed and get attached to the macromolecular network present on the small intestinal surface (Fig. 5d1). On the large intestinal surface, *B. infantis* cell clusters concentrated around the craters, where they were protected from washing steps. This phenomenon can be observed in Fig. 5d2. These characteristics of EPS and cluster formation together might contribute to a good adhesion efficiency of *B. infantis*. This is partially correlated with the results of the agglomeration test reported in section 3.1, where the agglomeration index of *B. infantis* ranked the 2nd or 3rd for SI and LI mucus, respectively, among the 5 strains tested. However, the adhesion *B. infantis* to SI mucus as reported in section 3.2, appeared quite low compared to the other strains. This could be due to the fact that *B. infantis* cells aggregated together, so that the number of colonies might therefore be lower than the number of single cells actually present in the sample.

Fig. 5e1, e2, e3 show the SEM images of *B. longum* adhered to the porcine intestinal surface. Although it is defined as the same species of *Bifidobacterium longum,* and differing only in the sub-strain, *B. longum* exhibited a very different adhesion behavior from that of *B. infantis*. *B. longum* cells, unlike *B. infantis*, did not aggregate into clusters but into pairs of two cells and were sparsely distributed over the intestinal surface. On the surface of the large intestine, *B. longum* cells were found scattered around the craters, (see Fig. 5e2). The low adhesion of *B. longum*, as observed by SEM, could be an explanation for the low agglomeration and adhesion to intestinal mucus, as reported in sections 3.1, 3.2, respectively.

It is important to note that SEM observation cannot comprehensively explain the adhesion phenomenon as it exhibits only the observed fields, which are very small. However, it somehow reveals the role of pili on the bacterial cell surface as well as the formation of long chains and clusters on bacterial adhesion to the intestinal surface [45,46].

## 4 Conclusions

This study provided a better understanding of the key factors influencing bacterial adhesion in the intestinal tract. The ability of bacteria to adhere to the mucus layer represents the first step to facilitate their colonization in the gut. The adhesion potential varies based on the cell surface properties of each bacterial strain. Besides, the morphological attributes of different strains also play a crucial role. Although imaging techniques can not provide a comprehensive investigation on the adhesion of bacteria to the intestinal surface, they can help to identify critical factors affecting the adhesion phenomena. The ex-vivo investigation by SEM reveals that the chain or cluster-forming ability of *F. duncaniae* and *B. infantis* could possibly facilitate bacteria to attach better to the specific receptors as well as the macromolecular network of the extracellular matrix scaffold in the mammal intestinal lining. Besides, the presence of EPS can also be an important factor in favor of the formation of biofilms and increases the adhesion potential of probiotics in the gut.

Moreover, this study contributes two *ex-vivo* approaches to study the bacteria – mucus agglomeration and the viable adhesion to mucus which are transposable in any conditions as they do not require any specific instruments. In both methodologies, the use of fresh mucus allows for consideration of the influence of the entire complex mucus system on bacterial cell interactivity. The viable bacteria-mucus adhesion assay considers only the impact of viable bacterial cells. The CFU assay allows for the enumeration of cultivable cells. This is an outstanding advantage of this approach compared to other methods, owing to the fact that cultivability is a fundamental requirement for bacterial interaction and proliferation within the intestinal tract. Compared to other *in-vivo* techniques that rely on indirect analysis through stool inspection, this approach distinguishes itself as the most direct methodology for analyzing bacterial interactions with gut components. However, before applying the viable adhesion assay, there should be an additional step to separate single cells from their aggregates. Based on these two proposed approaches, the results show that *E. coli* and *L. plantarum* exhibit the highest adhesion potential to mucus. In contrast, strains such as *F. duncaniae* and *B. longum* show lower mucus adhesion ability than the remaining strains. The difference in bacterial adhesion results between the two methods is possibly caused by the live or dead state of the bacteria. These results revealed that there might be an impact of the viable status of bacteria on their adhesion. Therefore, the effect of bacterial viability on their cell surface properties and thereby on the interaction in the intestinal tract should also be investigated.

To gain a deeper understanding of the bacterial adhesion mechanism to the intestinal tract at the molecular level, further experiments should be performed to investigate the physicochemical properties of bacteria and mucus layer. Moreover, considering bacteria and mucus as colloidal particles, extended theory like DLVO could also be applied to evaluate their adhesion potential.

## CRediT authorship contribution statement

**Thị-Thanh-Trúc Phùng:** Writing – review & editing, Writing – original draft, Visualization, Validation, Software, Methodology, Investigation, Formal analysis, Data curation, Conceptualization. **Sébastien Dupont:** Writing – review & editing, Methodology, Investigation, Funding acquisition. **Laurent Beney:** Writing – review & editing, Methodology, Investigation. **Sylvie Moundanga:** Writing – review & editing, Methodology. **Emmanuel Denimal:** Writing – review & editing, Software. **Phú-Hà Hồ:** Writing – review & editing. **Thomas Karbowiak:** Writing – review & editing, Writing – original draft, Visualization, Validation, Supervision, Project administration, Methodology, Investigation, Funding acquisition, Formal analysis, Data curation, Conceptualization.

## Declaration of competing interest

The authors declare that they have no known competing financial interests or personal relationships that could have appeared to influence the work reported in this paper.

## Figures and Tables

**Table:** Agglomeration of bacteria with intestinal mucus referred to as bacteria – mucus agglomeration index. For each bacterial strain placed in contact with fresh mucus, the experiments were performed on normalized concentration. The Latin letters a, b and c distinguish the difference between the 5 strains for their agglomeration ability with small intestinal mucus using one-way ANOVA with Tukey's test (p < 0.05). Similarly, the Latin letters a', b' and c' refer to their agglomeration ability with the large intestinal mucus. The difference between the agglomeration of each strain with SI and LI mucus was further analyzed by the student's t-test (p < 0.05). Corresponding results are indicated by the Greek letters α and β, followed by a number distinguishing each of the 5 strains.

	Agglomeration with small intestinal mucus (%)	Agglomeration with large intestinal mucus (%)
*Lactiplantibacillus plantarum*	48.10 ± 2.84^a,α1^	54.10 ± 1.07^a',β1^
*Escherichia coli*	82.54 ± 2.67^b,α2^	65.99 ± 3.44^b',β2^
*Faecalibacterium duncaniae*	12.06 ± 0.66^c, α3^	13.38 ± 0.62^c',α3^
*Bifidobacterium infantis*	52.58 ± 6.10^a,α4^	23.84 ± 9.08^c',β4^
*Bifidobacterium longum*	8.05 ± 0.80^c,α5^	14.47 ± 0.95^c',β5^

**Table:** Adhesion of viable bacteria to intestinal mucus layer coated on microtiter plate referred to as viable bacteria – mucus adhesion index. The Latin letters a, b and c distinguish the difference between the 5 strains for their adhesion to small intestinal mucus using one-way ANOVA with Tukey's test (p < 0.05). Similarly, the Latin letters a', b' and c' refer to their adhesion to the large intestinal mucus. The difference between the adhesion of each strain to SI and LI mucus was further analyzed by the student's t-test (p < 0.05). Corresponding results are indicated by the Greek letters α and β, followed by a number distinguishing each of the 5 strains.

	Adhesion to small intestinal mucus (%)	Adhesion to large intestinal mucus (%)
*Lactiplantibacillus plantarum*	2.18 ± 0.10^ab,α1^	2.54 ± 0.42^a'b',β1^
*Escherichia coli*	0.97 ± 0.24^c,α2^	1.76 ± 0.28^a',α2^
*Faecalibacterium duncaniae*	1.72 ± 0.80^bc,α3^	0.83 ± 0.19^b',α3^
*Bifidobacterium infantis*	0.66 ± 0.04^a,α4^	2.91 ± 0.75^a',β4^
*Bifidobacterium longum*	0.89 ± 0.01^ab,α5^	0.85 ± 0.31^b',α5^

**Figure:**
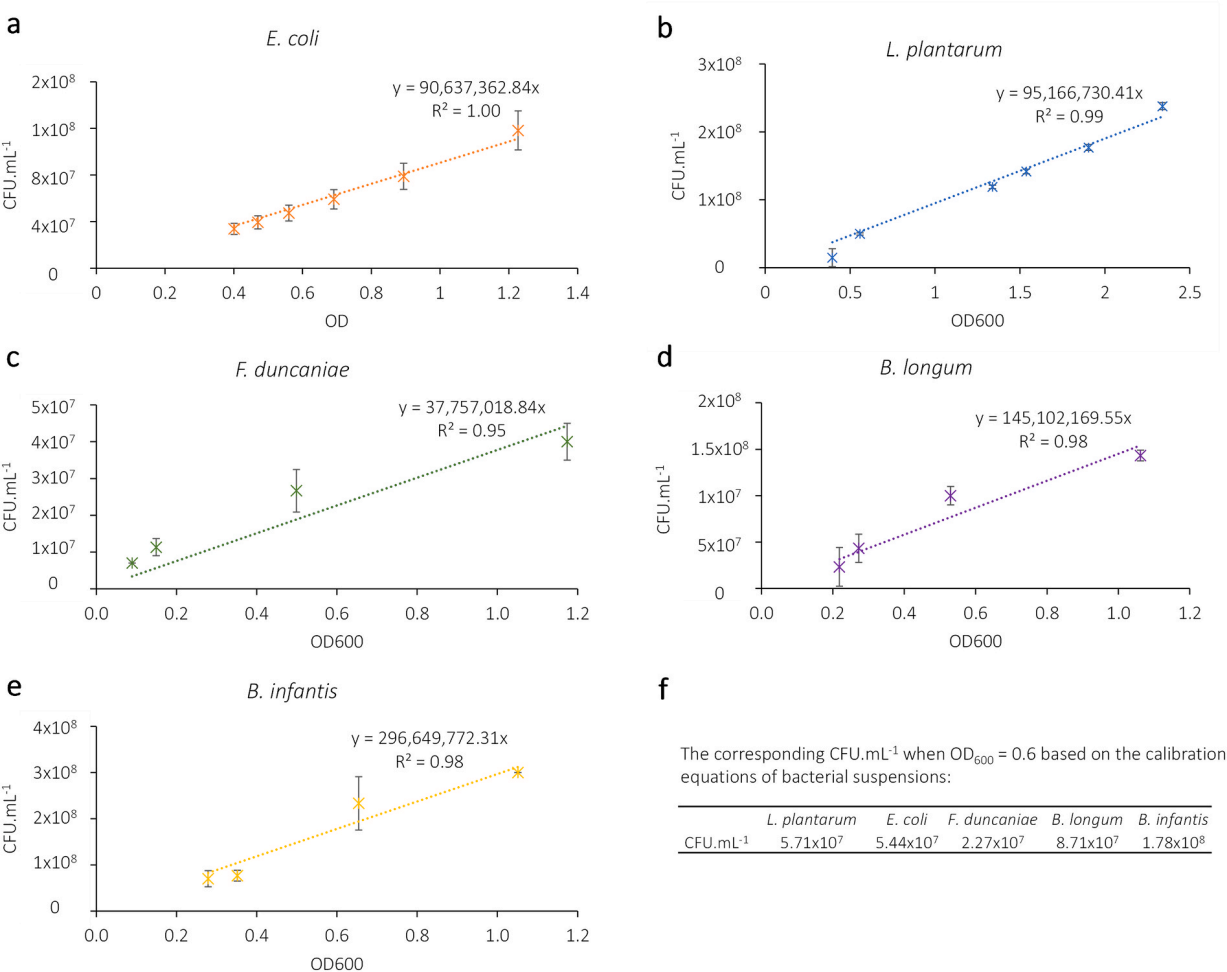
The calibration curves of OD_600_ vs. the corresponding CFU.ml^-1^ of 5 bacterial strains: *Lactiplantibacillus plantarum*, *Escherichia coli*, *Faecalibacterium duncaniae*, *Bifidobacterium infantis* and *Bifidobacterium longum*.
